# Factors Associated with Dual Practice in Surgery Specialists: Application of Multi-Level Analysis on National Registry Data

**Published:** 2019-05

**Authors:** Mahboubeh BAYAT, Roghaye KHALILNEJAD, Ali AKBARI-SARI, Iraj HARIRCHI, Gholamhossein SALEHI ZALANI, S. Elmira MIRBAHAEDDIN, Mahmoud KHODADOST, Ebrahim JAFARI POOYAN, Mehdi YASERI, Azad SHOKRI

**Affiliations:** 1. Center for Health Human Resources Research & Studies, Ministry of Health and Medical Education, Tehran, Iran; 2. Gerash University of Medical Sciences, Gerash, Iran; 3. Health Management and Economics Research Center, Iran University of Medical Sciences, Tehran, Iran; 4. Department of Health Management and Economics, School of Public Health, Tehran University of Medical Sciences, Tehran, Iran; 5. Department of Surgery, School of Medicine, Tehran University of Medical Sciences, Tehran, Iran; 6. Telfer School of Management, University of Ottawa, Ontario, Canada; 7. Department of Epidemiology, School of Public Health, Shahid Beheshti University of Medical Sciences, Tehran, Iran; 8. Department of Epidemiology, School of Public Health, Iran University of Medical Sciences, Tehran, Iran; 9. Department of Health Management and Economics, School of Public Health, Tehran University of Medical Sciences, Tehran, Iran; 10. Department of Epidemiology, School of Public Health, Tehran University of Medical Sciences, Tehran, Iran; 11. Social Determinants of Health Research Center, Research Institute for Health Development, Kurdistan University of Medical Sciences, Sanandaj, Iran

**Keywords:** Surgery specialists, Dual practice, Multiple jobs holding, Iran

## Abstract

**Background:**

Dual practice by surgery specialists is a widespread issue across health systems. This study aimed to determine the level of dual practice engagement and its related factors among Iran’s surgery specialists.

**Methods:**

A pre-structured form was developed to collect the data about surgery specialists worked in all 925 Iran hospitals in 2016. The forms were sent to the hospitals via medical universities in each province. The data were merged at the national level and matched using medical council ID codes, national ID codes and eventually a combination of the first name, surname and father’s name. Multilevel logistic regression was used to assessing the association between dual practice with study variables.

**Results:**

Overall, 14931 surgeons were participated **(**93% response rate) and 6405 (57% of) engaged in DP on total. Urinary tract & genital and neurosurgery specialties had the highest rank with 69%. DP was more frequent in specialists with higher age and experience, populated provinces, higher deprivation, and share of private hospitals. Faculty physicians (OR=0.69), full-time geographic physicians (OR=0.17), specialists with more than 25 years’ experience (OR=2.59) and age more than 40 yr (OR=1.3) had significant association with dual practice.

**Conclusion:**

Multi-approach strategy is needed to control dual practice through tax regulations, income cap, and limitations in work hours and number of visits in private sector.

## Introduction

About 30% of total burden of diseases require surgery for treatment and/or disease control (1) and 25% of mortalities related to diseases requiring surgery (2). Moreover, WHO has emphasized on this point that low and middle-income countries (LMICs) should deliver their surgery service as a cost-effective component of primary service delivery in order to reach millennium development goals (MDGs). Countries’ inability in this regard leads to significant prevalence of diseases and mortalities relating to surgery care (3). Gap in urgent surgery services may lead to death and/or inabilities resulting from treatable surgery cases, for instance, road accidents, burns and domestic injuries, infections, pregnancy-related complications, congenital anomalies, and acute abdominal conditions.

Moreover, need for such services has been on an increasing trend, population growth and aging, increased life expectancy, increased obesity prevalence, advancements in technology and surgery methods/procedures, especially non-invasive ones, and faster recovery from surgery was among contributing factors to escalated demand of surgery services (4). Older people need more medical services than young people. About 11% of a population who were over the age of 65 accounted for 40% of hospital dismisses and 48% of admission days (5). The more a population needs services, the more problems a health system confronts in order to provide adequate number of surgery services. According to WHO 80% of required infrastructures, including health workforce, to provide surgery services worldwide is available to less than 30% of urban population. While most people live in rural areas, lack of surgery specialists in rural areas may create a base for service delivery by non-specialists (2, 6).

One of major issues that affect supply and availability of surgeons in healthcare systems of different areas is their dual practice (DP). Dual practice is a state in which a physician is occupied simultaneously in both public and private sectors. However, level of DP engagement varies in different specialty areas. Surgery areas of specialties have shown higher DP engagement and based on a study in Denmark highest level of DP is witnessed among ear, nose and throat (ENTs) specialists, ophthalmologists, orthopedics and anesthetics (7). This phenomenon has been widely criticized because of its potential adverse effects on efficiency, effectiveness and quality of services and physicians performance and also due to decreased level of service delivery after their supply in public service. Physicians engaged in DP inadequately participated in public sector owing to ignorance of assigned duties or allocation of some of their public sector working hours to private sector. Some NHS surgeons change their performance in public sector to earn from private sector resources (8–10). Doctors engaged in DP were likely to perform more medical treatments than doctors who did not have a concurrent job in the private sector (11).

There was a correlation between increasing waiting time for receiving treatment in government/public hospitals and physicians DP (12). In Alberta, Canada, waiting list of cataract surgeons whose surgeries were financed by insurance companies and engaged in DP were longer than that of surgeons who worked only in the public sector (13).

Although identifying activity trend of surgeons is a key factor in the related planning in terms of total workforce supply and service provision, few studies have been published about dual practice engagement of surgery specialties. Thereby, this study was conducted to examine extent of this phenomenon in Iran and its distribution by different surgery specialties and different geographic regions of Iran. It also elaborated on various affecting/contributing factors.

## Materials and Methods

### Data collection process

A pre-structured form together with a manual about how to complete it was developed to collect the data about specialists worked in Iran public and private hospitals in 2016. Study hospitals were all 925 Iranian hospitals including government teaching hospitals, hospitals from social security organization (SSO), armed forces, Ministry of Petroleum (MoP) and other public, private and charity hospitals.

**Fig. 1:**
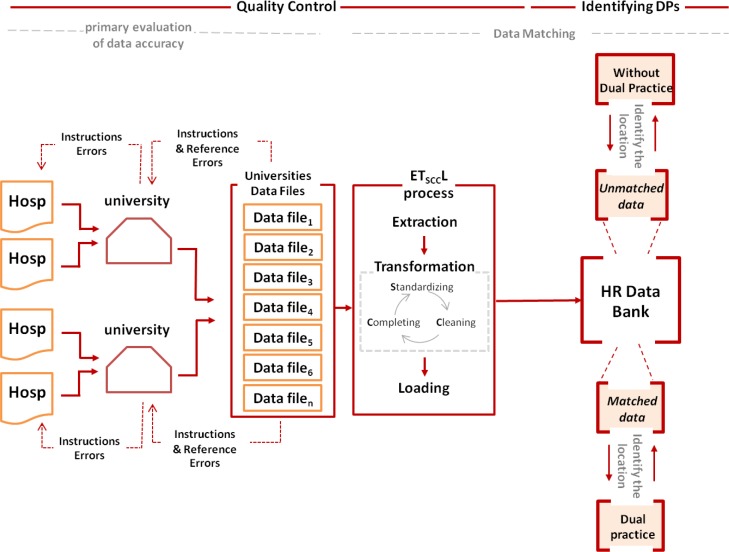
Data extraction process to identify medical specialists DP engagement

In each province of Iran, there is at least a government medical university directed by Ministry of Health and Medical Education (MOHME) that is responsible for education (training of medical doctors, specialists, nurses, etc.) and health services delivery. These medical universities have their own healthcare facilities but at the same time, they supervise all other public and private healthcare facilities in their catchment areas. Therefore, the forms were sent to all medical universities via MOHME and the medical universities sent the forms to all hospitals in their catchment areas. Medical universities then collected the data from hospitals of their catchment areas and sent them to MOHME using a pre-structured excel format that allowed integration of the data (Fig. 1).

The forms and the excel files included information about specialists’ name, surname, father name, national ID code, medical council ID code, socio-demographic characteristics, type of specialty, recruitment and job status, specifications of the employing hospitals and affiliating organization. The data were collected from each hospital for all surgery specialists worked in that hospital in 2016, full time or part time, temporarily or permanently. The hospitals were given one month to collect the data. For hospitals with no reply, a reminder at the end of month one and another reminder at the end of month two were sent. A combination of criteria from each individual specialist was used to merge the data at the province and national level and to identify whether a specialist was involved in DP. In the first step, the individual specialists’ medical council ID code was used to match and merge the data. If this ID code was not available, the national ID code was used; and eventually, if none of them was available for an individual, a combination of the first name, surname and father’s name or their initials were used. These procedures were performed by SQL functions of Access database (Table 1).

**Table 1:** Reference banks and their data items for completion of collected data

***Reference Banks***		***Data Items in the Reference Bank***
Medical Council	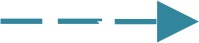	Medical council code, type of specialty, sex, age
MOHME human resource management office	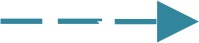	Faculty membership, experience, main occupation location
MOHME hospital management office	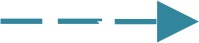	Full time status and experience
Medical Council Office permit/License	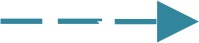	Office status
List of Clinics in Iran	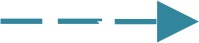	Name of clinic & its affilation

### Data Quality Control

After receiving information from each hospital, via medical universities, the following steps were performed.

1. Preliminary assessment of data accuracy: The completed forms and excel files could potentially have two types of errors; “Instructions Errors” spotting through corresponding data with the provided instruction form and “Reference Errors” detected by matching a number of fields with the reference data banks that were already available at MOHMO in terms of authenticity and accuracy. In case of having either of these errors, submitted forms were returned to the hospitals for correction and/or completion.
2. To minimize possible errors and increase accuracy of data collection and data merging a standard Extraction, Transformation and Loading (ETL) method was adopted and used (14). Separate data banks were developed for the MOHME (headquarter, schools/research centers, hospitals and clinics), private sector organizations (hospitals, clinics and physician offices) and other public sector organizations (hospitals and clinics). During Transformation, the generated data banks entered a cycle of standardizing, cleaning and completing.
  ○ Standardizing: Based on the assessment, errors of the data in the Excel software were detected and corrected. They include misspellings, heterogeneity in naming and heterogeneity in structure of the information.
  ○ Cleaning: Accuracy and precision of the data were confirmed using recaptured data in data matching for each standardized data item with the reference banks.
  ○ Completing: in this part, incomplete items consisting of faculty status, type of cooperation, demographic characteristics, etc. were completed through matching the data banks if needed. Moreover, one of the main objectives of this stage was completion of medical council codes for all records to proceed to the next stage which was identification of duplicate data between different extracted databases (15).

Finally, in loading stage, required information for the study objective were extracted and refined from different sources and then loaded into one main concentrated data bank (Table 1).

### Extraction of physicians with dual practice

Noting the nature of dual practice and attendance of physicians in more than one service delivery location, to identify these types of physicians a data matching model was applied and therefore duplicate data of medical council codes were detected among the health ministry banks and other public and private banks.

After identifying duplicate data (indicating physicians with dual practice), their main occupation location were specified based on their type of recruitment relation listed in the forms. Afterward, share of dual practice among public sector specialists in each province and its relation with other characteristics of the physicians and conditions of provinces were determined based on dual practice definition made by this study considering DP as employment of public sector physicians in private sector and other dissimilar public sectors in terms of ownership.

### Ethical Approval

This study was part of a PhD thesis (registration number 9223482001) that was supported by Tehran University of Medical Sciences Ethics Committee as IR.TUMS.VCR.REC.1395.1045 dated 16, November 2016.

### Statistical analysis

Study characteristics were reported using descriptive statistics and chi-square test. To take into account the hierarchical structure of our data, we used multilevel logistic regression analysis with three levels such as measured individual, districts and province factors to examine the relationships between DP and its contributory factors. The multilevel logistic regression analysis was performed using the xtmelogit command in Stata 14 software (Stata Corp, Texas, USA).

## Results

### Status of the study Surgery Specialists

Overall, 26597 records were collected for 14931surgery specialists from 858 (93%) hospitals out of total 925 hospitals from which 11223 specialists were considered based on the definition of this study. The mean age of specialists was 47.67 ± 9.54 and 65% of them were male. 82% of specialists were employed in medical university hospitals and 74% of all specialists were non-faculty members, and 27% of all specialists were employed on a contract basis. Moreover, the largest number of specialists in surgery group were gynecologists (24.3%) and the least number were neurosurgeons (3.8%) (Table 2).

**Table 2:** Demographic characteristics, University faculty membership, recruitment relation and main occupation location of the surgery specialists

***Variables***		***#specialists***	***%specialists***
Sex	Male	7,230	64.56
	Female	3,969	35.44
Age groups(yr)	40>	2,579	22.98
	40–45	1,713	15.26
	45–55	4,669	41.60
	55–65	1,632	14.54
	65<	630	5.61
Main occupation location	University hospital	9,220	82.15
	Social Security hospital	1,140	10.16
	Army forces hospital	309	2.75
	Petrochemical Company hospital	116	1.03
	Other public hospital	353	3.15
	MOHME headquarter	85	0.76
Faculty membership status	Non Faculty member	8,312	74.06
	Faculty member	2,911	25.94
Full-time employment status	Non-full-time	7,686	68.48
	FTG*	3,537	31.52
Recruitment relation	Permanent	3,012	26.84
	Zarib K	1,762	15.70
	Payam avar	11	0.10
	Peymani (semi-permanent)	1,166	10.39
	Contractual	3,086	27.50
	Other	1,338	11.92
	unspecified	848	7.56
Specialty groups	General Surgery	2,127	18.95
	Orthopedics	1,175	10.47
	Urinary tract and genital surgery	720	6.42
	Neurosurgery	429	3.82
	Nose and Throat and Head and Neck Surgery	796	7.09
	Obstetrics and Gynecology	2,724	24.27
	Ophthalmology	971	8.65
	Anesthesiology	2,281	20.32

* Full-time Geographic (FTG) physicians are the ones not allowed to be active in any other locations/sectors except their main occupation location.

**Fig. 2:**
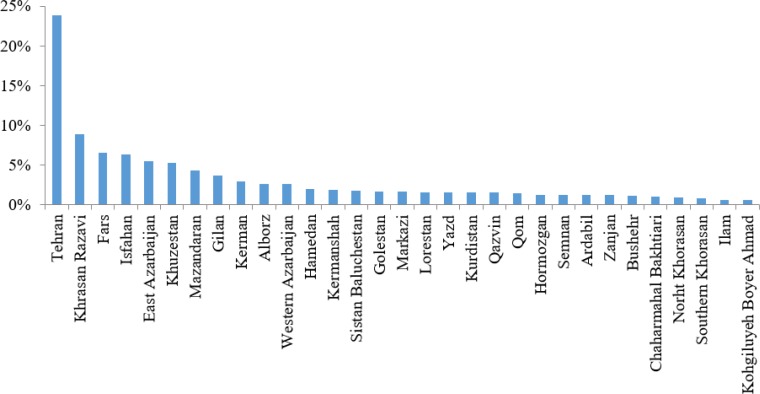
Distribution of surgery specialties in the provinces of Iran

23.78% of specialists were in Tehran (Fig. 2). Kohgiluyeh-Boyer Ahmad, Ilam, South Khorasan, and North Khorasan each had less than 1% of specialists (Fig. 2).

### Dual Practice Status

**Fig. 3:**
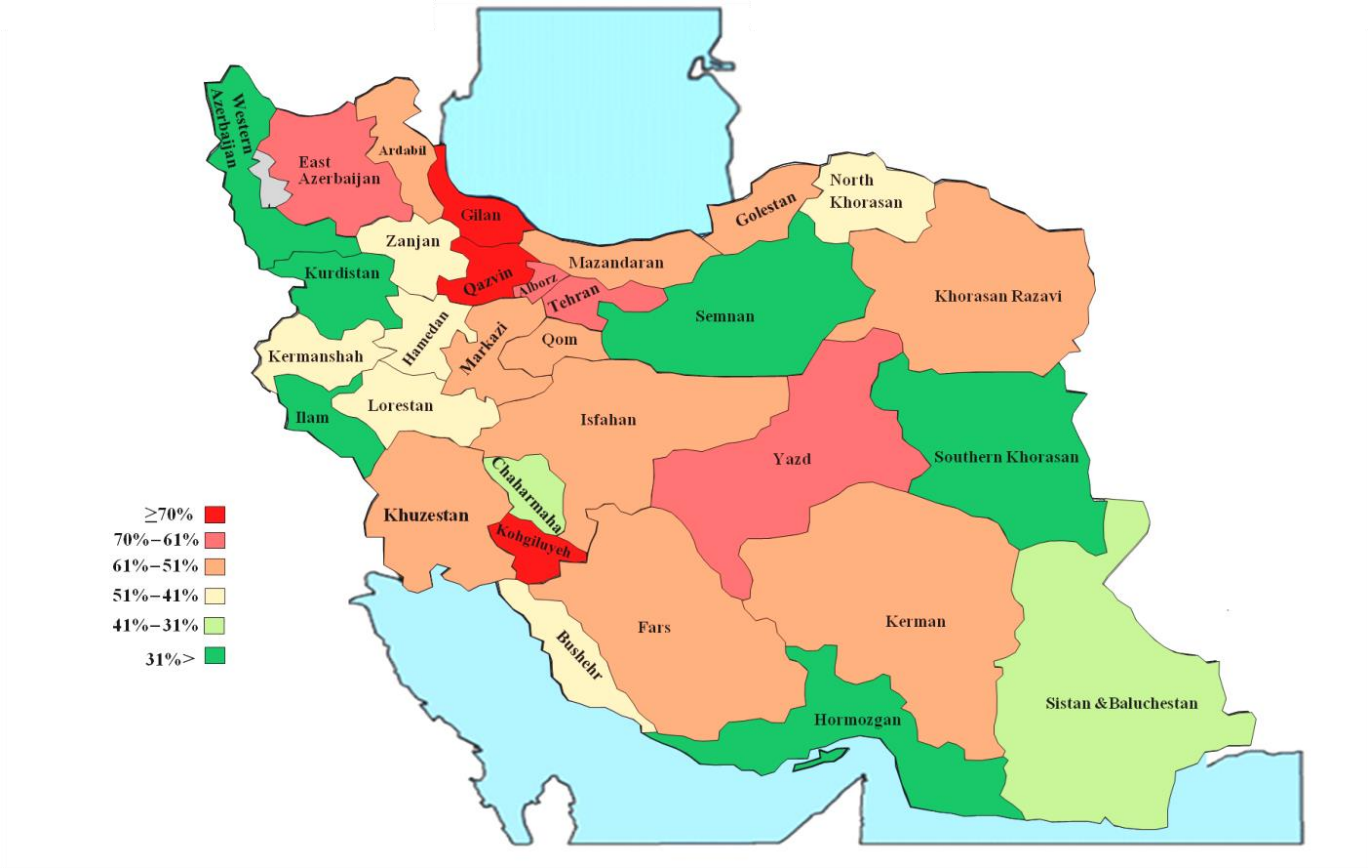
Map of geographical distribution of Iran’s surgery specialists engaged in dual practice

Overall, 6405 (57%) public sector surgery specialists were engaged in dual practice from which 5060 were in service through MOHME and 70% of non-full time specialists had multiple job holdings. Of 8312 Non-Faculty member specialists, 58% had multiple job holdings. As our findings show urology and neurology surgeons with 69% DP engagement had the highest proportion (Table 3). Based on this geographic distribution map which illustrates DP distribution in six groups, the highest rate of DP occurred in the provinces of Kohgiluyeh-Boyer Ahmad (80%), Gilan (79.6%), Qazvin (73%),Tehran (66 %), Alborz (65.9%), East Azerbaijan (65%) and Yazd (63%); compared to the provinces of Ilam, Semnan, Hormozgan, Southern Khorasan, West Azerbaijan, Kurdistan, Sistan and Baluchestan and Chaharmahal and Bakhtiari that had the lowest DP rates (Fig. 3, Table 3).

**Table 3:** Dual practice status of specialists by Affiliated Organization, faculty membership status, full-time recruitment and specialist physicians

***Variable***		***Total***	
		*#*	*%*
Affiliated Organization			
	MOHME	5,060	54.38
	Other public organs	1,345	70.13
Faculty membership			
	Non Faculty member	4,815	57.93
	Faculty member (academic)	1,590	54.62
Full time status			
	Non full-time	5,386	70.08
	FTG	1,019	28.81
Specialist Physicians			
	General Surgery	1,201	56.46
	Orthopedics	762	64.85
	Urinary tract and genital surgery	500	69.44
	Neurosurgery	295	68.76
	Nose and Throat and Head and Neck Surgery	506	63.57
	Obstetrics and Gynecology	1,792	65.79
	Ophthalmology	576	59.32
	Anesthesiology	773	33.89
Total dual practice		6,405	57.07

### Factors contributing to Dual Practice

The results of multilevel logistic regression show that there was no significant relationship between gender and dual practice engagement of surgery specialists (OR=1.04; 95%CI: 0.95–1.13). In contrast, faculty physicians to non-faculty ones (OR=0.69; 95%CI: 0.62–0.76) and full-time geographic physicians to non-full time physicians had less chance of DP engagement (OR=0.17; 95%CI: 0.15–0.19). Specialists with the age 40 yr and more, individuals with more than five years of job experience and physicians with Contractual and Permanent public sector employment relation had also more DP (*P*<0.05). In surgery specialists, urology had the highest (OR=1.85; 95%CI: 1.52–2.25) and anesthetics had the lowest (OR=0.35; 95% CI: 0.31–0.40) for DP engagement (Table 4).

**Table 4:** Multilevel logistic regression for assess the association between study variables and dual practice engagement of public sector specialists

***Variable***		***Odds Ratio (OR)***	***Confidence interval95%***		P-Level^*^	P-variable^**^
			***lower limit***	***upper limit***		
**Physicians Characteristics**						
** Sex**	Male	1.00				
	Female	1.043	0.958	1.135	0.328	0.328
** Age(yr)**	≤39	1.00				
	39–45	1.337	1.169	1.529	0.001	***0.001***
	45–55	1.738	1.556	1.943	0.001	
	55–65	4.099	3.510	4.786	0.001	
	65<	2.108	1.726	2.573	0.001	
** Experience**	≤5	1.00				***0.001***
	5–15	1.178	1.037	1.338	0.012	
	15–25	3.361	2.883	3.917	0.001	
	25<	2.597	2.144	3.146	0.001	
** Recruitment Relation**	Permanent	1.00				
	Zarib K	0.292	0.252	0.338	0.001	***0.001***
	Payam avar	0.130	0.026	0.640	0.012	
	Peymani (Semi-permanent)	0.451	0.390	0.521	0.001	
	Contractual	1.185	1.058	1.326	0.003	
	Others	0.913	0.791	1.054	0.215	
	Unspecified	0.292	0.252	0.338	0.001	
** Faculty membership status**	Non Faculty	1.00				
	Faculty	0.691	0.627	0.762	0.001	***0.001***
** Full-time status**	Non FTG	1.00				
	FTG	0.175	0.159	0.193	0.001	***0.001***
** Specialist Physicians**	General Surgery	1.00				
	Orthopedics	1.475	1.257	1.730	0.001	***0.001***
	Urinary tract and genital surgery	1.856	1.527	2.257	0.001	
	Neurosurgery	1.684	1.329	2.134	0.001	
	Nose and Throat and Head and Neck Surgery	1.337	1.115	1.602	0.002	
	Obstetrics and Gynecology	1.644	1.448	1.868	0.001	
	Ophthalmology	1.142	0.967	1.348	0.119	
	Anesthesiology	0.357	0.313	0.406	0.001	
**Provincial Characteristics**						
** Population (000)**	≤500.000	1.862	1.378	2.516	0.001	
	500–2.000	2.451	1.048	5.736	0.039	***0.001***
	2.000–5.000	3.647	1.359	9.788	0.01	
	5.000<	1.862	1.378	2.516	0.001	
** Extent of regional deprivation**		1.878	1.243	2.838	0.003	***0.003***
** Share of private hospitals**		1.506	1.196	1.896	0.001	***0.001***

* The *P*-value to assess the statistical significance of every level of multi-level categorical variables.

** The *P*-value for assess the overall statistical significance of a multi-level categorical variables

However, there was a significant increase in DP with the increase of population and in populations of 2000000–5000000 DP engagement has the highest chance (OR=3.64; 95%CI: 1.35–9.78). In addition, a 10% increase in the share of private hospitals is correlated with 1.50 times more DP (OR=1.50; 95%CI: 1.19–1.89). There was also an inverse correlation between regions’ deprivation and DP. Each unit of reduction in regional deprivation is correlated with 1.88 times less DP (OR=1.87; 95%CI: 1.24–2.83).

## Discussion

Findings of the present study showed that 6405 (57% of) specialists in surgery groups of Iran engaged in dual practice. In the meantime, the specialties of urology, neurosurgery, and Gynecology showed the highest DP rate. In Australia, surgery, gynecology and anesthesia specialties had the highest DP engagement (16). In Norway, DP highest rates were amongst ophthalmology (23.1%), ear, nose and throat & head and neck surgery (21.4%), orthopedic surgery (16.2%), general surgery (13.2%) and anesthesia (12.6%) (7). Ophthalmology, ear, nose and throat & head and neck surgery had a higher chance of engaging in DP. This shows difference in demand in private sector for surgery specialists compared to others in different countries. In fact, the earned income of these specialists in private sectors increased the chance of dual practice (17, 18). In South Africa, specialists with higher incomes showed more engagement in dual practice and they were typically surgeons (18). In UK private sector, neurosurgeons and ophthalmologists had the highest level of income while anesthetists had the lowest in the surgery group. The neurosurgeons had a higher income from the public sector rather than private. Therefore, physicians with greater salaries showed more engagement in DP (19).

Considering two facts; the banning law on dual practice in Iran health system for full-time geographic specialists (those under Zarib K and Payam Avaran contracts) and also the financial benefits of being a full-time employee in public sector (20), these specialists have a significantly lower chance of engaging in DP. However, a part of them sill engages in dual practice despite the prohibiting laws. In a study on surgery groups, non-full time physicians were more active in private sector comparing to full-time peers in private sector aiming to earn additional income from private sector (19, 21). This finding was consistent with the present study. Our results showed that faculty specialists had a lower chance of DP than their non-faculty peers. Different controlling mechanisms including financial and ranking incentives and etc. for the faculty specialists (20) has reduced their tendency to pursue dual practice. Albeit, DP engagement chance was higher amongst faculty specialists with higher ranks (7). Higher experience and skill among surgeons increased their chance of engaging in DP. Capacity and competency of experienced physicians provide them the means towards dual practice. While young physicians with less work experience prefer to work full-time in public sector (22). Moreover, senior physicians can develop and expand their career through DP and even learn new techniques in other medical areas while it is not feasible by working merely in public sector (23, 24).

On the other hand, private sector uses various encouraging mechanisms to attract experienced physicians (25). Therefore, senior physicians welcome higher income and engagement with private sector even if it is legally banned. In other words, low payment to senior physicians leads to their presence in private sector (26). In regions where private sector is highly attractive for physicians, applying total ban regulations puts high costs on public sector to maintain the senior physicians (22). Existence of service delivery units and inpatient facilities together with surgery capacities for performing surgeries in private hospitals have increased the tendency of surgeons to engage in dual practice with private sector (24, 27). Regions with higher demographic and human development index had higher chance of DP engagement (28). Likewise to the present study, central regions of Iran and those at vicinity of Tehran, the capital city, showed higher dual practice.

Comparing to remote and borderline regions, these areas are more developed and they have more population hence there are more equipped facilities (29). Therefore, considering developed facilities of private sector and variety of visits in advantaged areas, there are more employment opportunities in the private sector with higher income leading to increased employment in these areas (28).

## Conclusion

Despite slight decline in the chance of DP engagement in the surgery group though applying total ban rules and fulltime benefits, dual practice is still prevalent among them. Continuation of this trend among senior surgeons leads to waste of costs, increased inter-sectorial immigrations and finally drainage of public hospitals from experienced physicians in advantaged areas. Therefore, considering the urgent/pressing need for surgery groups in public sector to provide universal access to these services a multi-approach strategy is needed to control DP. This might include tax regulations, income cap, and limitation in work hours and number of patients visited/admitted in private sector.

## Ethical considerations

Ethical issues (Including plagiarism, informed consent, misconduct, data fabrication and/or falsification, double publication and/or submission, redundancy, etc.) have been completely observed by the authors.
